# Supplementary material for: Competition and dual users in complex contagion processes
Source: Sci Rep. 2018 Oct 1;8:14580. doi: 10.1038/s41598-018-32643-4 (PMC6167365; doi:10.1038/s41598-018-32643-4)
Supplement: Supplementary file 1 — Supplementary Information [file 41598_2018_32643_MOESM1_ESM.pdf]

# Supplementary Information

## Competition and dual users in complex contagion processes

**Byungjoon Min**[1,2*] **and Maxi San Miguel**[2,†]

[1]Department of Physics, Chungbuk National University, Cheongju, Chungbuk 28644, Korea
[2]IFISC, Instituto de Física Interdisciplinar y Sistemas Complejos (CSIC-UIB), Campus Universitat Illes Balears, E-07122 Palma de Mallorca, Spain
[*]bmin@chungbuk.ac.kr
[†]maxi@ifisc.uib-csic.es

## ABSTRACT

In this Supplementary Information (SI), we present first-order cascading condition for independent model and theoretical results for different parameter sets.

## 1 First-order cascading condition for independent model

The stability of the fixed point of recursion equations when $\rho_B \to 0$ gives a first-order condition for global cascading.[1–3] Applying the stability analysis to the independent model on ER graphs in which the degree distribution follows asymptotically the Poisson distribution, the condition reduces into $\langle k \rangle \Gamma(\lfloor 1/\theta_B \rfloor - 1, \langle k \rangle) = 1$ where $\lfloor . \rfloor$ denotes the floor function and $\Gamma(x,y)$ is the incomplete gamma function. More accurate cascading condition beyond the first-order approximation can be obtained by the maximum of NOI of recursion equations as noted in the main text.

## 2 Result for different parameter sets

We explore the final fraction of $R_A$, $R_B$, and $R_{AB}$ for the compatible and exclusive models with a different set of parameters, $\theta$, $\rho$, and $\langle k \rangle$ in order to verify how robust the role of dual users is. The final fraction of nodes in each state $A$, $B$, and $AB$ for the compatible and the exclusive model in the $\theta_A$ - $\theta_B$ plane is shown in Fig. 1. The role of dual users facilitating the spread of $B$ and the extinction of $A$ results in enlarged phase **B** in the compatible model comparing to the exclusive model. Thus, it shows again the effect of dual users mitigating the first-mover advantages of $A$. Next, we show that $R_A$ and $R_B$ with respect to $\rho_A$ and $\theta_B$ for $\theta_A = 0.1$ (Fig. 2), $\theta_A = 0.3$ (Fig. 3), and $\theta_A = 0.4$ (Fig. 4). Finally, we also study the effect of the initial fraction of $B$ with $\rho_B = 0.5, 1, 2$ (Fig. 5). We find that our main results consistently maintain for a broad range of parameters.

## References

1. Watts, D. J. A simple model of global cascades on random networks. Proc. Natl. Acad. Sci. **99**, 5766-5771 (2002).
2. Gleeson, J. P. & Cahalane, D. J. Seed size strongly affects cascades on random networks. Phys. Rev. E **75**, 056103 (2007).
3. Brummitt, C. D., Lee, K.-M. & Goh, K.-I. Multiplexity-facilitated cascades in networks. Phys. Rev. E **85**, 045102(R) (2012).

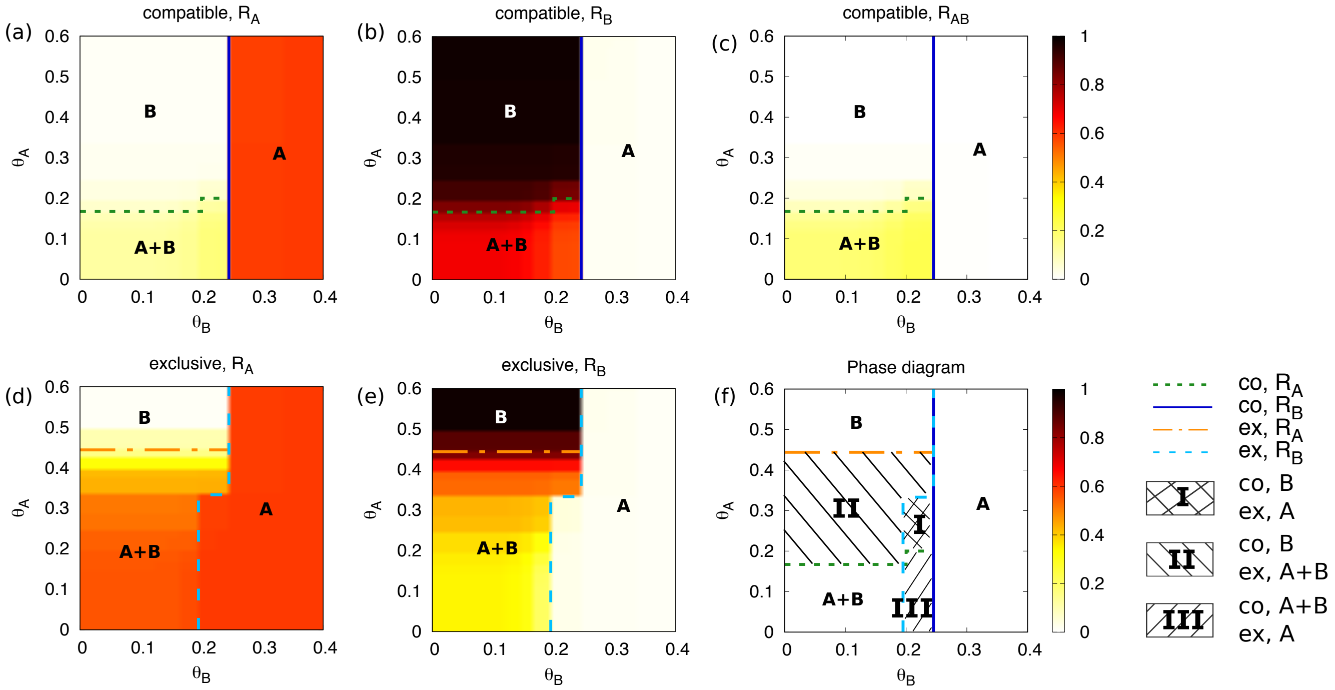


**Figure 1.** The final fraction of (a) $R_A$, (b) $R_B$, and (c) $R_{AB}$ for the compatible model and (d) $R_A$ and (e) $R_B$ for the exclusive model and (f) phase diagram with respect to $\theta_A$ and $\theta_B$ with $\langle k \rangle = 4$, $\rho_A = 0.2$, and $\rho_B = 0.01$. The transition lines for $R_A$ and $R_B$ for the compatible and exclusive models are shown together.

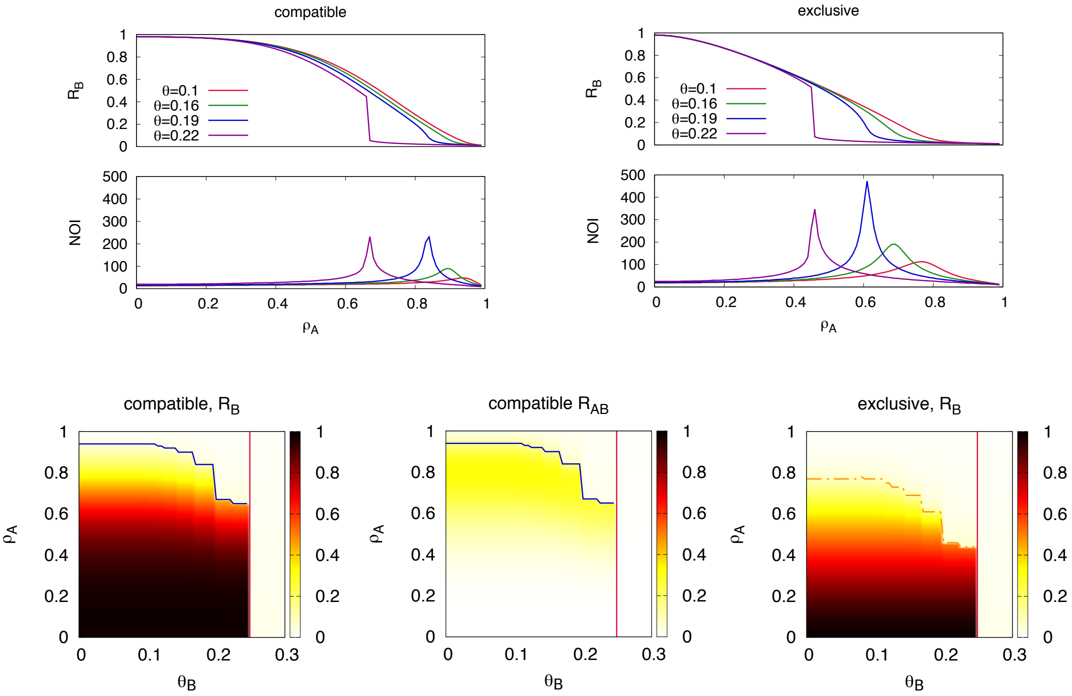


**Figure 2.** The final fraction of $B$ and NOI for the compatible and exclusive models by varying $\rho_A$ on ER networks with $\langle k \rangle = 4$, $\rho_B = 0.01$. $R_B$ and $R_{AB}$ with respect to $\rho_A$ and $\theta_B$ are shown with $\theta_A = 0.1$.

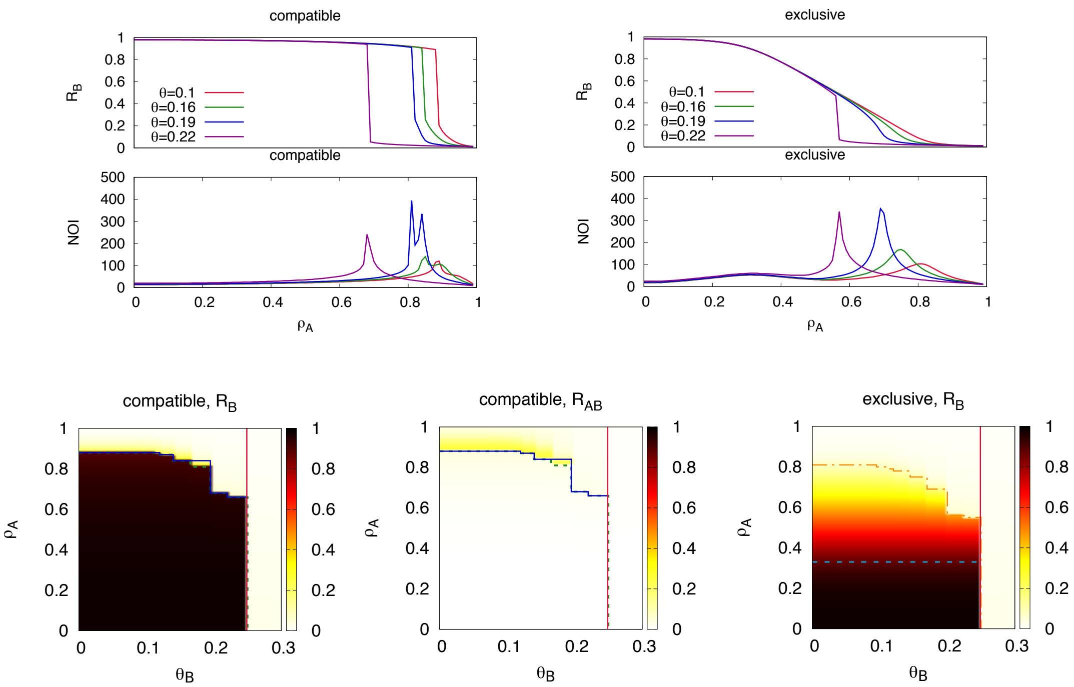


**Figure 3.** The final fraction of $B$ and NOI for the compatible and exclusive models by varying $\rho_A$ on ER networks with $\langle k \rangle = 4$, $\rho_B = 0.01$. $R_B$ and $R_{AB}$ with respect to $\rho_A$ and $\theta_B$ are shown with $\theta_A = 0.3$.

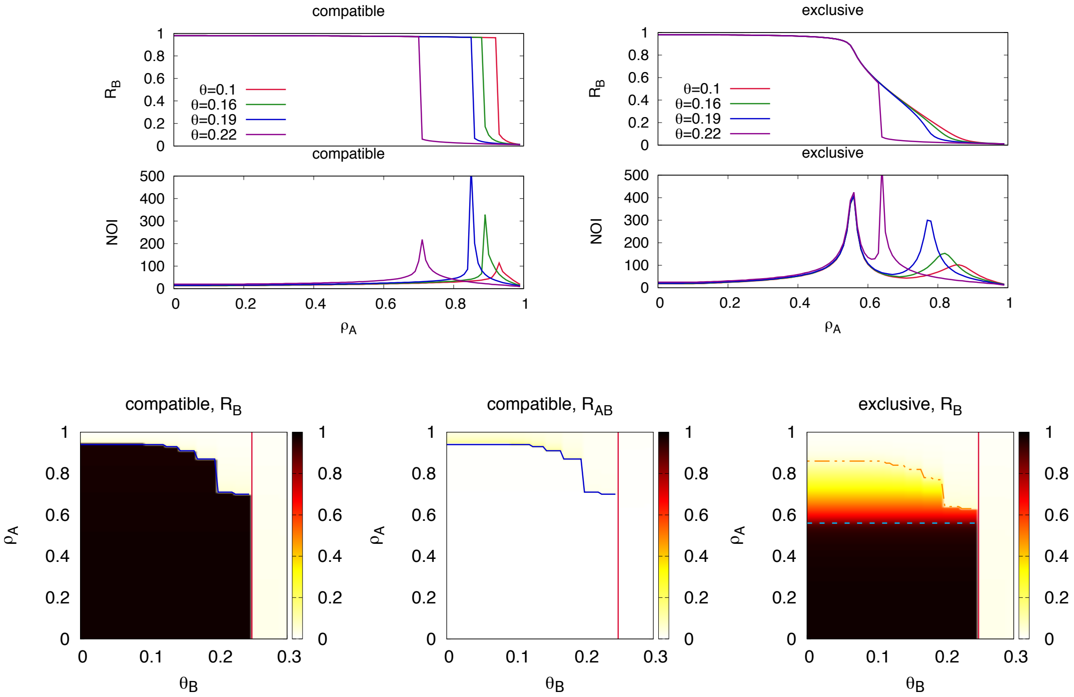


**Figure 4.** The final fraction of $B$ and NOI for the compatible and exclusive models by varying $\rho_A$ on ER networks with $\langle k \rangle = 4$, $\rho_B = 0.01$. $R_B$ and $R_{AB}$ with respect to $\rho_A$ and $\theta_B$ are shown with $\theta_A = 0.4$.

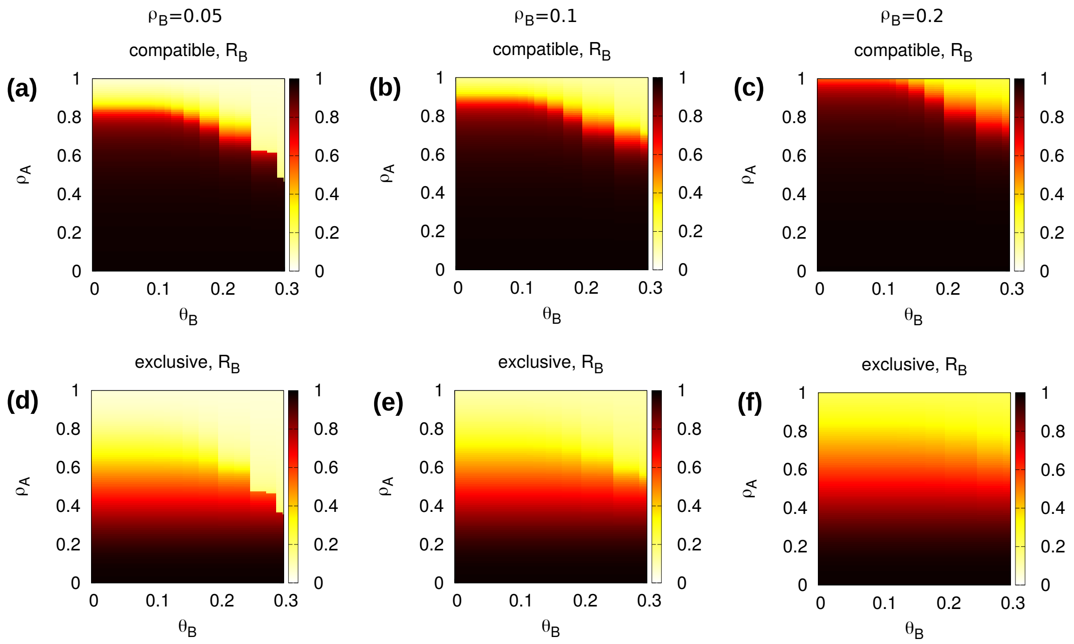


**Figure 5.** The final fraction of $B$ for the compatible and exclusive models on ER networks with $\langle k \rangle = 4$ with $\rho_B = 0.05, 1, 2$. $R_B$ with respect to $\rho_A$ and $\theta_B$ are shown with $\theta_A = 0.2$.
